# 962. Impact of Care Bundle Element Compliance on Clinical Outcomes for Pediatric Patients with *Staphylococcus aureus* Bacteremia

**DOI:** 10.1093/ofid/ofad500.023

**Published:** 2023-11-27

**Authors:** Raneem Pallotta, Karen Davidge, Daniel Riggsbee, Alison Tribble, Jerod Nagel

**Affiliations:** University of Michigan Medicine Health, Ann Arbor, MI; University of Michigan Health, Ann Arbor, Michigan; University of Michigan, Cement City, Michigan; C.S. Mott Children's Hospital, University of Michigan, Ann Arbor, MI; Michigan Medicine, Ann Arbor, MI

## Abstract

**Background:**

*Staphylococcus aureus* bacteremia (SAB) is a major cause of morbidity and mortality in children. The Infectious Disease Society of America (IDSA) outlines standards of care for patients with SAB known to positively impact morbidity and mortality, which have been validated in numerous studies within the adult population. However, there is limited data evaluating the clinical impact of compliance with these standards in pediatric patients. The primary objective of this study was to evaluate the impact of compliance with seven key bundle elements for the treatment of SAB in children.

**Methods:**

This was a retrospective, single-center, cohort study of pediatric patients aged 18 years or younger with SAB admitted to Michigan Medicine from 2019–2022. The seven bundle elements evaluated included timely initiation of antibiotics, appropriate targeted antibiotic therapy, ID consultation, documented culture clearance, an echocardiogram for high-risk patients, source control, and appropriate duration of therapy. To be considered fully compliant, patients had to meet all seven elements. The primary endpoint was a composite endpoint of mortality and 30-day infection-related readmission rates.

**Results:**

A total of 73 patients were included; 40 (54.8%) met the fully compliant bundle definition, and 33 (45.2%) were partially compliant. Fully compliant patients had a significantly lower incidence of combined mortality and readmission (7.5% vs. 24.2%; p = 0.0466). Lack of appropriate targeted therapy and lack of source control in patients with a removable source were the most common reasons for not meeting full bundle compliance (Table 1). Appropriate duration of therapy was met in 78.8% of patients in the partially compliant group (26/33, p = 0.0026).

**Table 1.**
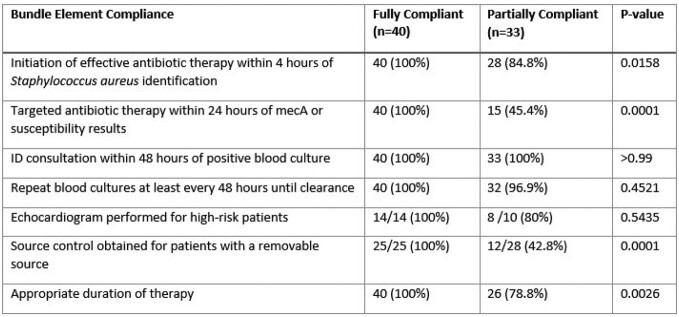
Bundle Element Compliance. Bundle element compliance between the two cohorts (fully bundle compliant vs partially bundle compliant)

**Table 2.**
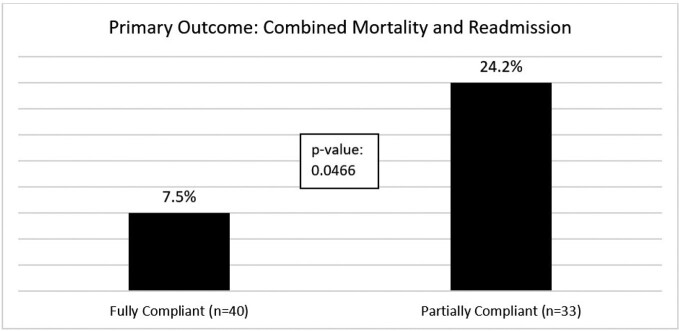
Primary Outcome

**Conclusion:**

Adherence to all bundle elements was associated with lower incidences of mortality and readmission. The results of this study will assist in quality improvement interventions at Michigan Medicine.

**Disclosures:**

**All Authors**: No reported disclosures

**Table 2.** Primary Outcome

Combine primary outcome of 30-day mortality and 30-day infection-related readmission (fully bundle compliant vs partially bundle compliant)

